# Role of Microbiota in the Skin Colonization of Candida auris

**DOI:** 10.1128/msphere.00623-22

**Published:** 2023-01-25

**Authors:** Brooke Tharp, Rachel Zheng, Garrett Bryak, Anastasia P. Litvintseva, Mary K. Hayden, Anuradha Chowdhary, Shankar Thangamani

**Affiliations:** a Department of Comparative Pathobiology, College of Veterinary Medicine, Purdue University, West Lafayette, Indiana, USA; b Mycotic Diseases Branch, Centers for Disease Control and Prevention, Atlanta, Georgia, USA; c Department of Internal Medicine, Division of Infectious Diseases, Rush University Medical Center, Chicago, Illinois, USA; d Medical Mycology Unit, Department of Microbiology, Vallabhbhai Patel Chest Institute, University of Delhi, Delhi, India; e National Reference Laboratory for Antimicrobial Resistance in Fungal Pathogens, Vallabhbhai Patel Chest Institute, University of Delhi, Delhi, India; f Purdue Institute for Immunology, Inflammation and Infectious Diseases (PI4D), West Lafayette, Indiana, USA; University of Georgia

**Keywords:** *C. auris*, horizontal transmission, skin microbiota, host defense, microbiome-based therapeutics

## Abstract

Candida auris is an emerging multidrug-resistant fungal pathogen that can cause life-threatening infections in humans. Unlike other *Candida* species that colonize the gut, C. auris efficiently colonizes the skin and contaminates the patient's environment, resulting in rapid nosocomial transmission and outbreaks of systemic infections. As the largest organ of the body, the skin harbors beneficial microbiota that play a critical role to protect from invading pathogens. However, the role of skin microbiota in the colonization and pathogenesis of C. auris remains to be explored. With this perspective, we review and discuss recent insights into skin microbiota and their potential interactions with the immune system in the context of C. auris skin colonization. Understanding microbiota, C. auris, and host interactions in the skin is important to develop microbiome-based therapeutic approaches to prevent and treat this emerging fungal pathogen in humans.

## PERSPECTIVE

Candida auris, an emerging multidrug-resistant fungal pathogen that predominately colonizes the skin, has been classified as an urgent threat by the U.S. Centers for Disease Control and Prevention (CDC) Antibiotic Threats Report (2019) and ranked in the critical priority group by the World Health Organization (WHO) in a recently released list of fungal priority pathogens (1, 2). C. auris is endemic at high prevalence in some long-term care facilities and acute care settings, where it can spread from patient to patient, resulting in outbreaks and systemic infections (3, 4). Several isolates of C. auris exhibit resistance to all three major classes of FDA-approved antifungal drugs, i.e., azoles, polyenes, and echinocandins. This poses a significant challenge to treat infections caused by this fungal pathogen (5, 6). Unlike most other *Candida* species, which colonize the gastrointestinal tract, C. auris efficiently colonizes the skin and contaminates the patient's environment (7), which may be related to C. auris’s unusual ability compared to other yeast to cause health care-associated outbreaks. Individuals colonized with C. auris can have a high fungal burden on their skin, which has been positively correlated with environmental contamination, transmission, and outbreaks of infections (7). Because skin colonization likely facilitates C. auris transmission and subsequent invasive disease, understanding the factors regulating C. auris skin colonization is critical to developing novel approaches to prevent and treat this emerging fungal infection in humans.

**FIG 1:**
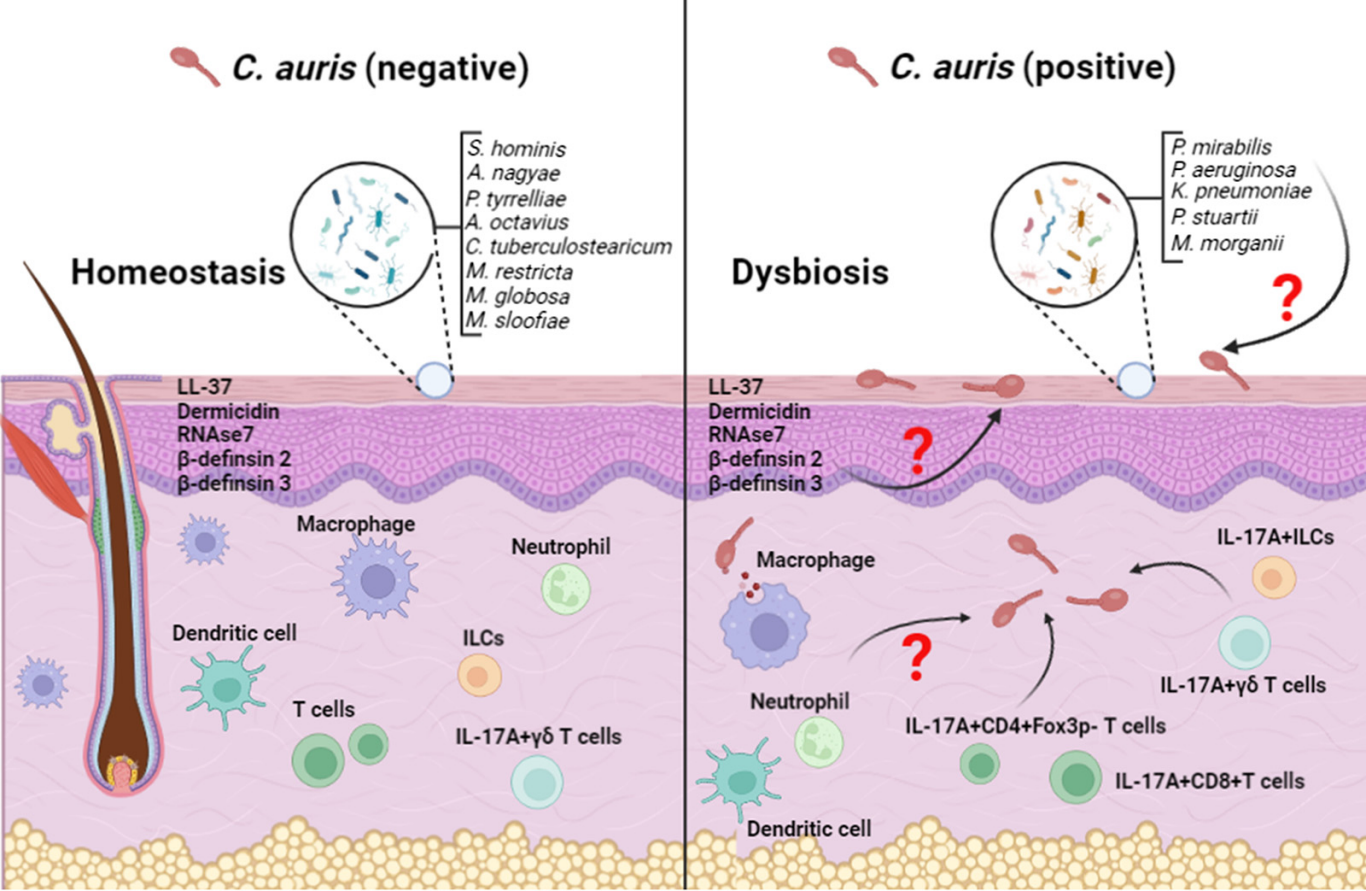
The figure illustrates the commensal microbiota and host factors in the C. auris-negative and -positive skin. The homeostatic portion (left) depicts a skin environment that has not been colonized by C. auris, while the dysbiosis (right) portion represents a skin that has been colonized by C. auris. Antimicrobial peptides such as LL-37, dermcidin, human β-defensin 2 and 3, and immune cells such as macrophages, neutrophils, ILCs, and dendritic cells that are integral to the skin immune defense against pathogens are shown in the C. auris-negative and -positive skin.

In human skin, C. auris coexists with commensal bacteria and fungi (3). Skin is colonized by a diverse set of commensal organisms (microbiota) that inhibit the growth and colonization of pathogens either by directly secreting small molecules and/or stimulating skin immune factors (8, 9). Results of this interaction can define the colonization level and pathogenesis of skin pathogens in humans. An interesting recent study by Proctor et al. investigated the associations between skin microbiota and C. auris colonization in humans (3). Utilizing 16S rRNA and internal transcribed spacer 1 (ITS1) of the rRNA gene locus sequencing approaches, bacterial and fungal communities were characterized using skin swabs from various body sites in both C. auris-positive and -negative individuals (Fig. 1). C. auris-negative individuals mainly harbored commensal bacteria such as Staphylococcus hominis, Staphylococcus epidermidis, Staphylococcus caprae, Anaerococcus nagyae, Peptoniphilus tyrrelliae, Anaerococcus octavius, and Corynebacterium tuberculostearicum. In contrast, C. auris-positive patients showed a different bacterial community, mainly containing Proteus mirabilis, Pseudomonas aeruginosa, Klebsiella pneumoniae, Providencia stuartii, and Morganella morganii. Fungal members also displayed a considerable shift in C. auris-positive individuals (3, 10). Fungal microbiota in C. auris-negative individuals were dominated by *Malassezia* species such as Malassezia restricta, Malassezia globosa, Malassezia furfur, and Malassezia arunalokei. On the other hand, the microbiota fungal community of C. auris-positive individuals was dominated by a mixture of *Candida* species, including C. auris, C. albicans, C. tropicalis, C. glabrata, and others (3, 10). The difference in both commensal bacteria and fungi communities present in C. auris-positive and C. auris-negative patients provides evidence that the host microbiome may play an important role in the colonization of C. auris on the skin. Although microbiota dysbiosis was observed in the skin of C. auris-colonized individuals, it is still not known whether alterations in the microbial community are a consequence of C. auris colonization or whether microbiota dysbiosis contributes to C. auris colonization in the skin.

Commensal microbiota induce innate and adaptive host immune factors to eliminate skin pathogens (11–13). To operate optimally, microbiota, and the immune system need to communicate effectively. Though the role of skin immune factors in C. auris skin colonization is rudimentary, recent studies indicate that skin immune factors such as LL-37 and interleukin 17 (IL-17) inhibit C. auris growth and skin colonization (14, 15). LL-37, a major antimicrobial peptide (AMP) expressed by human keratinocytes, inhibits C. auris growth *in vitro* (15). LL-37 showed fungistatic and fungicidal activity against C. auris at concentrations of 25 to 100 and 50 to 200 μg/mL, respectively. Furthermore, LL-37 caused fungal cells to undergo extensive surface changes, inhibited the cell cycle, and induced oxidative stress in C. auris (15). Mice express cathelicidin antimicrobial peptide (CAMP), a homologue of human LL-37 peptide. CAMP is regulated by the hypoxia-inducible factor 1 alpha (HIF-1α) transcription factor. Mice lacking CAMP peptide or HIF-1α alone in the skin are highly susceptible to skin bacterial pathogens (16–18). CAMP and HIF-1α are also highly expressed in the intestine, where they are necessary to provide gut microbiota-mediated resistance to C. albicans intestinal colonization (19). On the other hand, skin microbiota such as Staphylococcus epidermidis induce LL-37 (CAMP) in keratinocytes (20). Among three AMPs (LL-37, defensin-1, and defensin-2) induced by S. epidermidis, LL-37 expression was increased 100-fold compared to 10-fold induction of defensin peptides in treatment with S. epidermidis (20). However, currently, the role of microbiota-LL-37 interactions in C. auris skin colonization is not clear. Future studies, such as using CAMP knockout mice, are critical to understanding the role of skin microbiota and AMP regulation in C. auris colonization in the skin.

Another recent study by Huang et al. identified that epicutaneous infection with C. auris elicited different immune cell types (14) (Fig. 1). T helper 17 cells (CD4-positive [CD4^+^] IL-17A^+^ and CD4^+^ IL-17F^+^), IL-17A- and IL-17F-producing CD8^+^ T cells, IL-17A-producing γδ T cells, and IL-17A-producing innate lymphoid cells (ILCs) were significantly increased in the skin samples 14 days after infection. Using mice deficient in IL-17R-associated adaptor molecule Act1, which lacks IL-17 responses, the authors demonstrated that IL-17A/IL-17F response is associated with protection against C. auris. Significantly increased fungal load was observed in the skin samples of *Act1^−/−^* groups compared to wild-type mice. Further using *Rag2^−/−^* (which lacks T cells) and *Rag2^−/−^ II2rg^−/−^* (which lacks T cells and ILCs) mouse models, the authors identified that fungal load in skin samples from these mice was significantly increased, indicating the contribution of IL-17A/IL-17F produced by αβ T cells, γδ T cells, and ILCs to control C. auris colonization in the skin. On the other hand, skin microbiota such as S. epidermidis induces IL-17^+^ T cells to control cutaneous C. albicans skin colonization (21). However, given that host immune response to C. auris differs from C. albicans (22, 23) and there is a difference in immune response elicited by different commensal species of bacteria (21), findings from C. albicans and S. epidermidis cannot be readily extrapolated to C. auris and other microbiota species, respectively. Future studies are necessary to understand microbe-immune cell interactions in the context of C. auris infection.

Given that the skin of C. auris-positive individuals lacks several skin commensal bacteria present in the skin of C. auris-negative individuals (3), understanding the role and mechanisms through which skin microbiota regulate C. auris will expand the knowledge about the microbiota and host immune factors that control C. auris skin colonization. Restoring commensal microbiota in the C. auris-positive and/or individuals highly susceptible to C. auris colonization may be important in establishing a normal microbial ecosystem and preventing C. auris colonization of skin. An expansion of a diverse microbial community will enhance microbial diversity and immune defense that could prevent the selective growth of skin pathogens, including C. auris (11, 13, 24, 25). Furthermore, microbiota synergize with host immune factors to inhibit the colonization of skin pathogens (11, 13, 24, 25). Harnessing the immunomodulatory capacity of symbiotic factors from skin microbiota has a potential therapeutic role against C. auris. However, several questions remain to be answered (Fig. 1). How do skin commensals regulate C. auris colonization? Are the host AMPs (LL37, β-defensin 1, β-defensin 2, β-defensin 3, RNase 7, and dermidicin) and immune cells (innate and adaptive) involved in the defense against C. auris skin colonization? Identification of commensal microbes that regulate C. auris colonization will open the door for novel approaches to prevent C. auris skin colonization, subsequent nosocomial transmission, and mortality due to invasive C. auris infection in humans. For example, skin microbiota such as Staphylococcus hominis are currently in human clinical trials for the treatment of skin bacterial pathogens and atopic dermatitis (9, 12). This supports the feasibility of future interventions using commensal bacteria to prevent and treat C. auris skin colonization. Taken together, identification of bioactive microbial factors and the relevant host immune pathways regulated by specific microbiota will not only increase our understanding the microbiota-mediated mechanisms regulating C. auris skin colonization but also will provide a platform to develop novel antifungal therapeutics.
